# Patient experience after shared medical appointments and in-office WALANT carpal tunnel release: a prospective evaluation of satisfaction, cost and waste reduction

**DOI:** 10.1016/j.jpra.2026.08.017

**Published:** 2026-08-19

**Authors:** Mattias Hedspång, Simon Briland, Marcus Sagerfors, Daniel Reiser

**Affiliations:** a Department of Orthopedics and Hand Surgery, Faculty of Medicine and Health, Örebro University, SE Örebro, Sweden; b Department of Orthopedics and Hand Surgery, Örebro University Hospital, Region Örebro County, PO Box 1613, SE-701 16 Sweden

**Keywords:** Carpal tunnel release, WALANT, Shared medical appointment, Group consultation, In-office surgery, Patient experience, Cost-minimization, Surgical waste

## Abstract

**Purpose:**

To evaluate patient experience after a revised pathway combining same-day shared medical appointments with in-office wide-awake local anesthesia with no tourniquet (WALANT) carpal tunnel release, and to assess efficiency, waste generation, and costs.

**Methods:**

This prospective observational pilot study included patients undergoing carpal tunnel release using a revised pathway at a Swedish hand surgery department. Approximately six patients per half-day session attended a structured preoperative shared group information session before surgery, with an opportunity for individual questions before local anesthesia. Procedures were performed in two consultation rooms using WALANT, field sterility, limited draping, absorbable sutures, and simplified follow-up. No anesthesiologist or anesthesia personnel were involved. Patient experience was assessed with the Picker Patient Experience-15 (PPE-15) questionnaire and postoperative questions. Waste was weighed and a cost-reduction analysis was performed.

**Results:**

Sixty patients (mean age 55 years, 70% women) underwent 62 carpal tunnel releases. The mean PPE-15 score was 97.8, and 57 of 60 respondents reported no pain. Of the 17 patients with previous contralateral standard carpal tunnel release, 16 rated the revised pathway as better and one as equal. Five patients met the criteria for physician wound assessment; none had infection. Waste was reduced from 2.20 kg to 0.55 kg per procedure. The estimated saving was 225 Euro per procedure at the clinic level and 289 Euro at the regional level when reduced follow-up was included.

**Conclusion:**

Same-day shared medical appointments combined with in-office WALANT carpal tunnel release were associated with high patient satisfaction, low reported pain, reduced waste, improved efficiency, and lower estimated costs. Larger comparative studies are needed to confirm the safety and evaluate the independent effect of group information.

## Introduction

Carpal tunnel release is one of the most common procedures in hand surgery, and is increasingly performed under local anesthesia. In Sweden, the incidence of carpal tunnel release has increased over time, with substantial regional variation, reflecting both the high burden of carpal tunnel syndrome and the importance of efficient surgical pathways.^1^ The growing use of wide-awake local anesthesia with no tourniquet (WALANT) surgery has enabled selected hand surgery procedures to move from the conventional operating room (OR) to procedure rooms or office-based settings.^2^ This transition may improve access to care and reduce the need for anesthetic and OR resources including OR nurses, but it also raises an important patient-centered question: can a more efficient surgical pathway preserve, or even improve, the patient experience?

Patient information is a central component of safe and acceptable surgical care. Traditionally, preoperative information is delivered individually, often weeks or months before surgery or shortly before the procedure. Shared medical appointments and group-based education have been introduced in several areas of healthcare as a way to improve access, increase the time available for providing information, and allow patients to learn from the questions and concerns of others. Previous research suggests that shared medical appointments can improve patient trust, perceived quality of care, and patient satisfaction, although the format and outcomes vary between settings.^3^

Group preoperative education before elective surgery has also been associated with improved patient-centered outcomes including knowledge, activation, reduced anxiety, less postoperative pain, and some aspects of quality of life, although the evidence remains heterogeneous.^4^ Studies on group counseling for informed consent have shown mixed results, but emphasize the relevance of how information is delivered and understood in clinical and research settings.^5^ The COVID-19 pandemic highlighted the need to optimize the use of OR resources and OR nurses as well as the increasing need for procedures that can be performed under local anesthesia in-office.^6^, ^7^, ^8^ However, shared medical appointments have rarely been studied as an immediate preoperative consultation in hand surgery.

The aim of this study was to evaluate patient experience after a shared medical appointment combined with in-office WALANT carpal tunnel release. Secondary aims were to assess procedural efficiency, cost reduction, postoperative wound concerns, and the amount of waste generated compared with the standard pathway.

## Methods

### Study design and setting

This was a prospective study conducted at the Department of Orthopaedics and Hand Surgery, X University Hospital, Sweden. The study was approved by the Swedish Ethical Review Authority (registration number EPM 2024–04,742-01) and registered in the Swedish public registry of clinical trials. The health economic evaluation was performed from a healthcare provider perspective, as described in the health economic analysis plan.

### Participants

Individuals aged 18 years or older who were on the waiting list for carpal tunnel release at Örebro University Hospital were eligible for inclusion. Patients were recruited by telephone from the surgical waiting list by two of the authors. If a patient’s referral and clinical history were considered appropriate for direct booking to surgery without an additional preoperative outpatient visit, they were scheduled for the revised pathway.

Before inclusion, all patients completed a detailed health questionnaire, which was reviewed by the clinical team. Unclear responses, relevant comorbidities, or potential contraindications were addressed individually before the patient was accepted for the pathway. On the day of surgery, the operating surgeon verified the indication, relevant medical history, previous surgery, medication, allergies, and suitability for the procedure. Patients were also given the opportunity to raise personal concerns or provide additional information not included in the referral or questionnaire. If the available information was insufficient or the patient was considered unsuitable, surgery would be postponed and an individual outpatient assessment arranged. Written informed consent for participation in the study was obtained on the day of surgery, after the shared medical appointment and before administration of local anesthesia.

Each half-day session included approximately six patients. The session started with a 30-minute shared medical appointment led by the operating surgeon (experience level 3–4 according to Tang and Giddins). (Tang JB, Giddins G. Why and how to report surgeons’ levels of expertise. J Hand Surg Eur Vol. 2016;41(4):365e366.)

This revised pathway differed from our standard pathway for carpal tunnel release in several aspects, including the format and timing of preoperative information, the surgical setting, anesthetic technique, staffing, draping, postoperative dressing, and follow-up routine. The main differences between the revised pathway and the standard pathway are summarized in Table 1.

**Table 1:** Summary of differences between the revised and standard pathways'.

**Revised pathway**	**Standard pathway**
Preoperative 30-minute group consultation (with time for individual consultation before administration of local anesthesia)	Preoperative consultation months in advance or short consultation before the operation in cases with direct referral to surgery
Operation in office (2 rooms)	Operation in minor surgery operating room
6 operations in 3 h	5 operations in 4 h
Ordinary clothes for patients, plastic drape for surgeon	Special patient/surgical clothing and draping
Surgeon disinfects and operates without assistance; assistant nurse aids patients in and out of rooms	Surgeon assisted by operating nurse who also prepares and drapes the patient
WALANT surgery; buffered local anesthesia with epinephrine	Local anesthesia; bloodless field with pneumatic tourniquet
Self-adhesive soft silicone dressing with bandage; normal use of hand unless no pain	Large bulky dressing
Paper form for operational notes	Standard computer operation notes
Absorbable sutures	Non-absorbable sutures requiring removal
Follow-up only if patient suspicion of postoperative problems	Usually suture removal at primary healthcare center

**Fig. 1:**
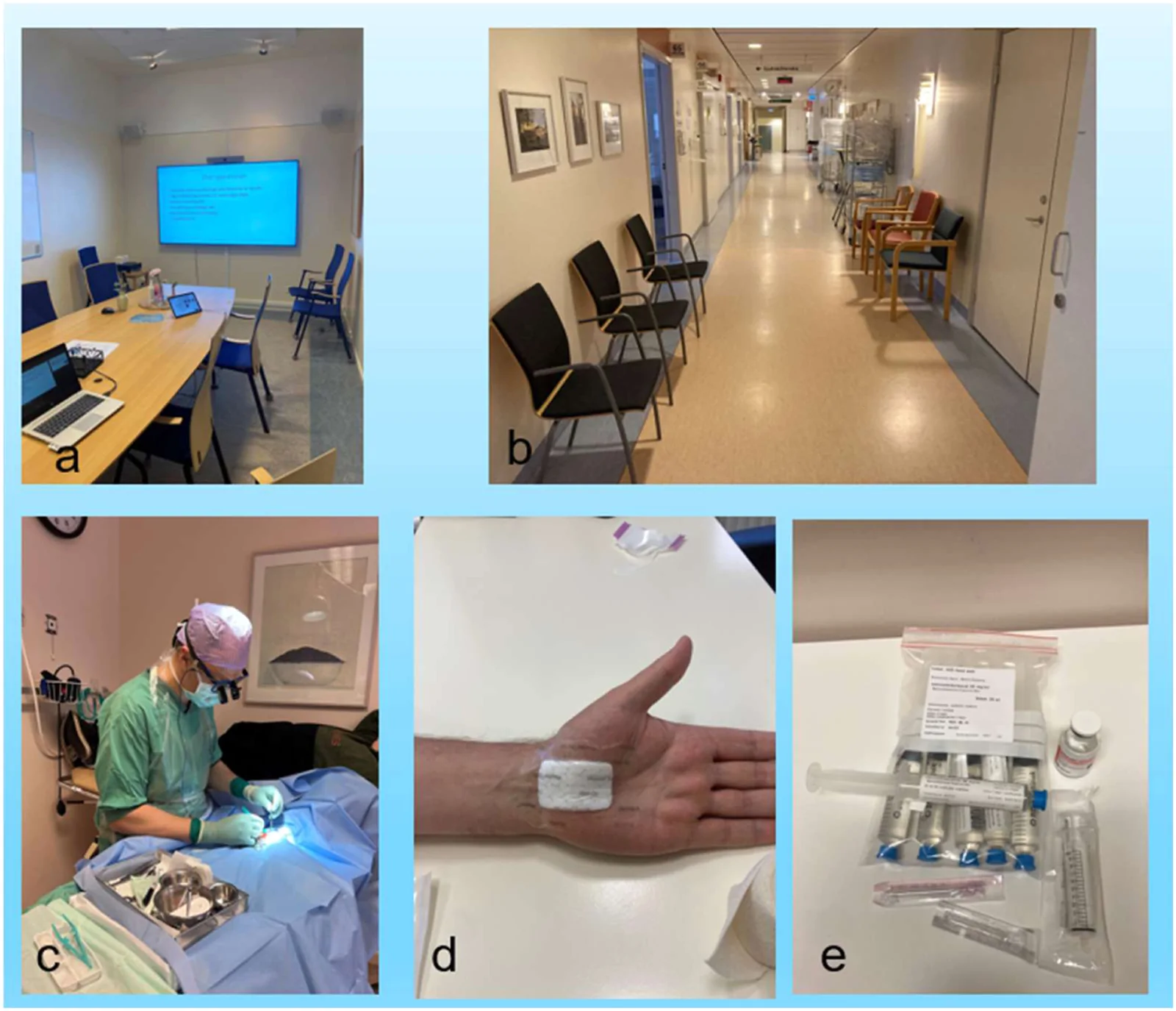
Overview of the revised pathway for in-office WALANT carpal tunnel release. **(a)** Room used for the preoperative shared medical appointment. **(b)** Waiting area outside the consultation rooms. **(c)** Setup for the in-office procedure, with the surgeon alternating between two standard consultation rooms. **(d)** Postoperative soft silicone dressing and bandage. **(e)** WALANT anesthesia using buffered local anesthetic with epinephrine.

The revised pathway included a preoperative group consultation with approximately six patients, use of two consultation rooms, no patient clothing change, limited draping, WALANT anesthesia, absorbable sutures, and selective rather than routine postoperative follow-up (Fig. 1).

During the group session, patients received structured information about carpal tunnel syndrome, the planned operation, the anesthetic method, expected postoperative course, wound care, hand use after surgery, and when to seek medical advice. The format allowed patients to hear questions raised by others and to receive the same core information together. After the group session, each patient also had the opportunity to ask individual questions before local anesthesia was administered by the surgeon. Written informed consent for participation in the study was obtained on the day of surgery, after the shared medical appointment and before administration of local anesthesia.”

The operations were performed in two standard consultation rooms rather than in a conventional OR. Buffered local anesthesia with adrenaline was administered in all patients before the first operation started. The procedure was performed using field sterility (Fig. 1c). Patients remained in ordinary clothes. The surgeon wore sterile gloves, cap, mask, and a plastic apron. Limited draping was used. Absorbable sutures and a self-adherent soft silicone dressing were applied. Patients were advised to use the hand normally within the limits of pain, provided that no analgesics were required.

The surgeon alternated between the two rooms. While one patient was being operated on, the next patient could be prepared in the other room. The surgeon performed disinfection, draping, and the operation without an operating nurse. One assistant nurse helped patients in and out of the rooms and supported the workflow. Patients were offered a postoperative nurse visit 10 to 14 days after surgery. The nurse assessed the wound according to predefined written criteria for possible infection: pus, increased pain, redness, or heat. If any of these criteria were present, a physician was asked to evaluate the patient. Decisions regarding diagnosis of infection, wound culture, and antibiotic treatment were recorded.

The standard pathway consisted of carpal tunnel release in a minor surgery OR, with an operating nurse assisting the surgeon, more extensive sterile draping, a pneumatic tourniquet, non-absorbable sutures, and routine follow-up for suture removal in primary care.

### Patient experience

Immediately after surgery, patients completed the Swedish version of the Picker Patient Experience-15 (PPE-15) questionnaire, unsupervised. The PPE-15 is a validated instrument for measuring a patient’s experience of the care they have received, and includes domains such as information, respect, involvement, pain, and support after discharge. In the present study, the questionnaire was used to assess patient experience of the revised surgical pathway.^9^^,^^10^

At the postoperative follow-up visit, patients answered additional targeted closed questions. Patients who had previously undergone carpal tunnel release on the contralateral hand using the standard pathway were asked whether they considered the revised pathway to be better, equal, or worse. They were also asked whether they considered the anesthetic method to be better, equal, or worse. Patients who had not previously undergone contralateral carpal tunnel release were asked whether they would be willing to undergo the same procedure again if needed.

### Waste measurement

To estimate waste generation, the total amount of waste produced during one day of operations using the revised pathway was weighed. On the following day, waste from three operations performed using the standard pathway was weighed. Mean surgical waste weight per operation was then calculated for each pathway .

### Cost-reduction analysis

A cost-minimization analysis was performed to estimate the difference in cost per procedure between the revised and standard pathways. The cost of standard carpal tunnel release was identified from the regional inter-county billing agreement for 2024. This analysis assumed that the two pathways differed in three main resource categories: personnel during the operation, materials used, and need for postoperative follow-up.

Material use was identified by comparing pick lists for the standard operation with direct observation of materials used during the revised pathway. Prices were retrieved from the regional supply service and adjusted to 2024 price levels using the Statistics Sweden consumer price index calculator. Personnel costs were calculated using the mean monthly salary, including social fees, for operating nurses in 2024, obtained from the regional HR department. The cost of postoperative follow-up was based on the regional price list. To avoid overestimating savings, the estimated need for follow-up in primary care was calculated conservatively by doubling the proportion of patients who met the criteria for physician evaluation at the postoperative nurse visit.

All costs were converted from Swedish krona to Euro using the mean exchange rate for 2024.

### Statistical analysis

Descriptive statistics were used. Continuous variables are presented as means or medians, as appropriate. Categorical variables are presented as numbers and percentages. No formal hypothesis testing was performed, as this was a prospective observational pilot study designed to evaluate feasibility, patient experience, and resource use.

## Results

A total of 60 patients underwent 62 carpal tunnel releases using the revised pathway. Their mean age was 55 years, and 42 of them were women (70%). Two patients underwent bilateral procedures on separate occasions. For these patients, responses from the second postoperative questionnaire were excluded from the patient-experience analysis to avoid repeated responses from the same individual.

The mean PPE-15 score was 97.8 out of a maximum possible score of 100. Most patients (57/60, 95%) reported no pain during the care episode. Among patients who had previously undergone conventional carpal tunnel release on the contralateral side, 94.1% considered the revised pathway to be better, while none considered it worse. Furthermore, 97.6% of patients without previous contralateral surgery stated that they would be willing to undergo the same pathway again if needed. One patient did not complete the full PPE-15 questionnaire and was excluded from the PPE-15 analysis. The patient underwent surgery on one side only.Two patients cancelled the postoperative follow-up visit; one of them completed the postoperative questionnaire by mail, but no wound assessment was performed (Table 2).

**Table 2:** Patient experience measured with the Picker Patient Experience-15 (PPE-15) questionnaire and postoperative evaluation.

**Variable**	**n/N (%)**
Patients (cases) included in PPE-15 analysis	59 (61)
Mean PPE-15 score (0–100)	97.8
Patientcases reporting no pain (PPE-15 question 10a)	57/61 (93.4%)
Patients with previous contralateral carpal tunnel release	17
Revised pathway rated as better	16/17 (94.1%)
Revised pathway rated as equal	1/17 (5.9%)
Revised pathway rated as worse	0/17 (0%)
Patients without previous contralateral surgery	41
Would choose the same pathway again if needed	40/41 (97.6%)
Would not choose the same pathway again	1/41 (2.4%)

Data are presented as n or n/N (%) as appropriate.

The median time to postoperative follow-up was 14 days. Five of 60 patients met the predefined criteria for evaluation by a physician at the postoperative follow-up visit. None of these patients were diagnosed with a surgical site infection, none had a wound culture taken, and none were prescribed antibiotics.

The mean weight of waste generated by the revised pathway was 0.55 kg per operation, compared with 2.20 kg per operation using the standard pathway. This corresponds to a 75% reduction in waste weight per procedure.

The cost-reduction analysis showed a calculated saving of 225 Euro per operation at clinic level. When reduced need for postoperative suture removal and wound check in primary care was included, the total estimated saving increased to 289 Euro per operation at the regional level. The main drivers of cost reduction were reduced operating-nurse time, reduced material costs, and fewer postoperative primary-care visits.

The detailed calculation estimated reduced material costs of 17 Euro per procedure and reduced personnel costs of 208 Euro per procedure, resulting in a clinic-level saving of 225 Euro. Reduced primary-care nurse visits contributed an additional estimated saving of 63 Euro, giving a total regional saving of 289 Euro per procedure (Table 3).

**Table 3:** Estimated savings per procedure when using the revised pathway.

Cost of carpal tunnel release according to the regional price list (converted from SEK)
Reduced material costs
Reduced personnel costs *(operating nurse salary including social security contributions = 6362 Euro; 160 h/month; standard pathway = 5 procedures per 4 h; study pathway = 6 procedures per 4 h)*
Saving at clinic level per procedure
Reduced primary-care nurse visits *(80% of visits avoided; one visit = SEK 856)*
Saving at regional level per procedure

## Discussion

The main finding of this study was that a revised pathway for carpal tunnel release, combining same-day shared medical appointments with in-office WALANT surgery, was associated with favorable patient experience scores, low patient-reported perioperative pain, reduced waste generation, increased procedural efficiency, and lower estimated costs. The pathway also enabled six procedures to be performed during a half-day session compared with five procedures using the standard pathway. Importantly, the model appeared to improve three areas that are often perceived as competing priorities in healthcare: patient experience, environmental sustainability, and cost efficiency.

The development of WALANT surgery has changed the logistics of hand surgery by reducing the need for anesthetic support, tourniquets, and conventional OR resources.^2^^,^^11^ Carpal tunnel release is particularly suitable for such pathways because it is a common, quick, and well-standardized procedure.^1^ Previous studies have shown that carpal tunnel release can be performed safely in minor procedure rooms using field sterility, with low infection rates,^12^ and office-based WALANT surgery has been associated with high patient satisfaction.^13^

Our findings are consistent with this literature. The mean PPE-15 score was 97.8, and 57 of 60 respondents reported no pain during the care episode. Among the 17 patients who had previously undergone standard contralateral carpal tunnel release, 16 rated the revised pathway as better and one as equal. No patient with previous experience of the standard pathway rated the revised pathway as worse. This suggests that the increased efficiency of the pathway did not come at the expense of patient acceptance.

A central component of the revised pathway was the structured preoperative group information. The group session was not merely an efficiency measure, but an integrated part of the patient-centered pathway. All patients received the same core information immediately before surgery, and also had the opportunity to ask individual questions before local anesthesia. In addition, patients were exposed to questions and concerns raised by other participants, which may have improved understanding and reassurance while ensuring that all patients received standardized information. In Sweden, there is no general statutory minimum waiting period between informed consent and this type of elective surgery. In healthcare systems where a mandatory waiting period applies, the information and consent process would need to be completed during a separate consultation before the day of surgery. The shared medical appointment on the day of surgery could then be retained to repeat the information, address additional questions, and confirm the patient’s willingness to proceed.

Shared medical appointments have previously been described as a model that may improve access, patient-clinician interaction, patient trust, and perceived quality of care.^3^ Group preoperative education has also been associated with improvements in several patient-centered outcomes including knowledge, activation, reduced anxiety, less pain, and some aspects of quality of life, although the evidence remains heterogeneous.^4^ Studies of group counseling in informed consent have shown mixed effects on comprehension, but underline the importance of how information is delivered and understood.^5^

Wong et al. evaluated shared medical appointments as a model for carpal tunnel surgery consultation and found them comparable to individual consultations regarding surgical risk recall, patient satisfaction, and comfort.^14^ In that study, however, the appointment was separated from the surgical procedure. Conversely, in our study the group information was delivered immediately before surgery and combined with in-office WALANT surgery in the same session. Although we did not measure the independent effect of the group information, the high patient-experience scores and strong patient preference among those who had experienced both pathways suggest that immediate group information is acceptable and could be a valuable component of efficient patient-centered care. The opportunity to hear questions raised by other patients may represent an important advantage of the group format, as it allows participants to benefit not only from the information provided by the surgeon but also from issues they may not have considered themselves.

Patient experience was measured using the Swedish version of the PPE-15, which was originally developed and validated for assessing patient experience in inpatient care.^10^ Patient characteristics may influence reported care experience,^9^ and so the very high PPE-15 scores in the present study should be interpreted with some caution. However, the specific responses regarding pain, willingness to undergo the same pathway again, and comparison with previous standard contralateral surgery support the overall interpretation that the pathway was well tolerated.

Healthcare contributes substantially to public-sector greenhouse gas emissions, and consumables are an important aspect of this impact.^15^ Operating rooms are recognized as major sources of healthcare waste, and hand surgery has been identified as a suitable field for waste-reduction initiatives because many procedures are short, frequent, and performed in a limited surgical field.^16^ Recent studies have demonstrated that procedure-room carpal tunnel release and lean surgical setups can reduce waste and carbon footprint compared with conventional OR pathways.^17^, ^18^, ^19^

In the present study, waste generation was reduced considerably, primarily through limited draping, avoidance of patient clothing changes, fewer disposable items, and the use of an office-based setting. Although we only measured waste weight and did not perform a formal carbon-footprint analysis, the magnitude of the reduction suggests that this type of pathway may provide environmental benefits. Our findings therefore support previous reports indicating that substantial reductions in surgical waste can be achieved without compromising patient experience or workflow efficiency.^16^, ^17^, ^18^, ^19^

The cost-minimization analysis demonstrated an estimated saving of 225 Euro per procedure at the clinic level and 289 Euro at the regional level when reduced postoperative follow-up was included. The principal cost drivers were reduced personnel requirements, lower material consumption, and fewer routine follow-up visits.

These findings are consistent with previous studies demonstrating that WALANT-based procedure-room surgery can reduce resource utilization while maintaining patient satisfaction.^17^^,^^18^ ^,^^13^ However, the exact magnitude of the savings should be interpreted within the Swedish healthcare context. Staffing models, OR costs, reimbursement systems, salaries, material prices, and follow-up routines differ considerably between healthcare systems, making direct comparisons difficult.

This study has some limitations. First, it was a prospective observational pilot study without a control group. Consequently, we cannot conclude that the revised pathway is superior to the standard pathway, although the subgroup of patients who had previously undergone contralateral surgery strongly preferred the revised model.

Second, the effect of the group consultation was not evaluated separately. It is therefore not possible to determine whether the observed patient satisfaction was primarily related to the shared medical appointment, the WALANT technique, the absence of a tourniquet, the office-based setting, the simplified dressing, or the overall workflow. Future studies should compare individual and group information within otherwise identical in-office WALANT pathways.

Third, the study was not powered to detect rare adverse events such as surgical site infection. No infections were observed, but larger studies are required to confirm safety. Finally, the PPE-15 was originally developed for inpatient care,^10^ the waste analysis was based on waste weight rather than carbon-footprint calculations, and the economic evaluation relied on local Swedish costs.

Several components of the pathway were changed simultaneously, including the format and timing of preoperative information, anesthetic technique, surgical setting, staffing, draping, suture material, dressing, and follow-up routine. Consequently, the present study cannot determine which individual component contributed to the favorable patient experience, and the findings should be interpreted as relating to the integrated pathway as a whole. The pathway was introduced as a complete clinical workflow rather than sequentially as an experimental evaluation of its separate components. Future comparative studies should evaluate individual components while keeping the remainder of the pathway unchanged.

## Conclusion

A revised pathway combining same-day shared medical appointments with in-office WALANT carpal tunnel release was associated with high patient satisfaction, low reported pain, no observed infections, reduced waste, increased procedural efficiency, and lower estimated costs. The findings suggest that patient-centered care, resource efficiency, and reduced waste can be combined in high-volume hand surgery. Further comparative studies are needed to evaluate the independent contribution of group information and to confirm safety, patient experience, and environmental impact in larger cohorts.

## Ethical approval

The study was performed according to the Helsinki declaration and approved from the Swedish ethics review authority (reference number 2024–04,742-01).

## Author’s contributions

MH study design, data collection and analysis and writing of the manuscript. SB study design, data collection and writing of the manuscript. MS study design and writing of the manuscript. DR study design, data analysis and writing of the manuscript. All authors have read and approved the final manuscript.

## Availability of data and materials

The datasets used and/or analysed during the current study are available from the corresponding author on reasonable request.

## Declaration of competing interest

The authors declare that they have no conflict of interest.
